# Bioengineering of bacteria for cancer immunotherapy

**DOI:** 10.1038/s41467-023-39224-8

**Published:** 2023-06-15

**Authors:** Dinh-Huy Nguyen, Ari Chong, Yeongjin Hong, Jung-Joon Min

**Affiliations:** 1 Institute for Molecular Imaging and Theranostics, Department of Nuclear Medicine, Chonnam National University Medical School and Hospital, Gwangju, 61469 Republic of Korea; 2 Department of Molecular Medicine (BrainKorea21 Plus), Chonnam National University Graduate School, Gwangju, 61469 Republic of Korea; 3 Department of Nuclear Medicine, Chosun University Medical School and Hospital, Gwangju, 61452 Republic of Korea; 4 Department of Microbiology, Chonnam National University Medical School, Gwangju, 61469 Republic of Korea

**Keywords:** Cancer, Cancer microenvironment, Cancer prevention

## Abstract

Here, we provide a brief overview of the approaches and strategies underlying bacteria-based cancer immunotherapy (BCiT). We also describe and summarize research in the field of synthetic biology, which aims to regulate bacterial growth and gene expression for immunotherapeutic use. Finally, we discuss the current clinical status and limitations of BCiT.

The relationship between cancer and human microbiota has existed since ancient times. However, bacteria-based cancer immunotherapy (BCiT) was not attempted until the 19^th^ century, when William Coley used live or heat-killed *Streptococcus pyogenes* and *Serratia marcescens* to treat patients with inoperable cancer. This strategy led to > 10-year disease-free survival in about 30% of the patients^1,2^. Several genera of facultative and obligate anaerobic bacteria, such as *Clostridium*, *Bifidobacterium*, *Listeria*, *Salmonella*, *Escherichia*, *Proteus*, and *Lactobacillus*, have been extensively studied because of their ability to specifically target and inhibit tumor growth^3^. Some of these bacterial species have been engineered to deliver drug payloads and improve their safety and targeting efficiency^4–6^. For example, several attenuated *Salmonella* strains were generated via deletion of major virulence genes (e.g., *S. typhimurium* defective in guanosine 5’-diphosphate-3’-diphosphate synthesis [the ∆ppGpp strain]), and showed that these bacteria localized to the tumor and reached over 1 × 10^10^ colony-forming units (CFU)/g in tumor tissue 3 days after intravenous injection; moreover, the tumor-to-normal tissue bacterial ratio exceeded 10,000:1^4,7,8^.

Bacteria have distinct ways of targeting and penetrating tumors, using unique properties such as their inherent motility, chemotaxis, and the ability to induce inflammatory reactions and evade the immune system^3^. Once colonized the tumor, bacteria expand within the immune-privileged tumor microenvironment (TME) to induce antitumor immunity by increasing immune surveillance and decreasing immunosuppression^3,9^. However, bacteria alone cannot eliminate tumors completely; thus the prospect of generating bacteria carrying therapeutic payloads (drugs) may hold great promise in cancer treatment^6^.

As a tumor-targeting drug carrier, bacteria can be genetically engineered to produce potent therapeutic payloads such as cytotoxic agents, immunomodulators, cytokines, prodrug converting enzymes, small interfering RNAs, and nanobodies^4^. These payloads and the bacteria work together to reprogram the TME by interacting with tumor cells, immune cells, and other TME components. This reprogramming is accomplished through the infiltration and activation of immune cells, as well as the production of cytokines and chemokines. Thus, the overall antitumor effect of the bacteria themselves is increased^4^.

Synthetic biology tools for genetic engineering enable control of bacterial activity within tumor tissues, thereby increasing the safety and efficacy of bacteria-based cancer therapy^4,6^. The development of synthetic gene circuits, which can sense and respond to specific signals, enables bacteria to precisely control payload production (Table 1). In this Comment, we outline the progress in the engineering of bacteria for cancer immunotherapy, focusing mainly on studies using *S. typhimurium* and *E. coli*, the most widely investigated strains in the context of genetic engineering and cancer immunotherapy.

**Table 1:** Example of bacteria-based cancer immunotherapy approaches

Bacteria strain	Promoter	Inducer	Therapeutic payload	Application	Reference
*S. typhimurium* ∆ppGpp	P_*BAD*_	L-arabinose	Pore-forming Cytolysin A (ClyA)	Killing cancer cells, which may enhance the antitumor immune response	^4,13^
			Flagellin B (FlaB)	Enhanced M2-to-M1 macrophage polarization	
*S. typhimurium* ∆ppGpp	P_*Tet*_	Doxycycline	ClyA and *Renilla* luciferase 8 (Rluc8)	Theranostic approach	^14^
*E. coli* Nissle 1917	P_*Dawn*_	Light	Tumor apoptosis-related inducing ligand (TRAIL)	Induction of tumor cell death, resulting in activation of antitumor immunity	^4^
*E. coli* Nissle 1917	P_*BV220*_	Temperature (Heat)	Tumor necrosis factor-α	Activation of immune responses against tumor	^17^
*E. coli* Nissle 1917	P_*LacI*_	Focused ultrasound	PD-L1- and CTLA-4-blocking nanobodies	Enhanced antitumor T cell immunity	^19^
*E. coli* Nissle MG1655	Acid-responsive promoter	Acid	ClyA	Killing cancer cells, which may enhance immune responses against tumors	^20^
*E. coli* Nissle 1917	Quorum-sensing P_*lux*_	Acyl-homoserine lactone (AHL)	CD47 nanobody (CD47nb)	Increased stimulation of tumor-infiltrating T cells targeting primary and metastatic tumors in animal tumor models	^26^
			PD-L1- and CTLA-4-blocking nanobodies	Strong activation of antitumor T cell immunity	^6,21,33^
*E. coli*	Two AND-gate promoters	AHL and IPTG	Luminescence	Dynamic changes in gene expression	^23^
*E. coli* Nissle 1917	Genome editing-P_*arg*_	NH_4_Cl	L-arginine	Enhanced antitumor activity of T cells	^6,21,34^

## Genetic engineering approaches for BCiT

In BCiT, genetic engineering is used to induce precise and effective antitumor responses. Various genetic circuits, comprising genes and promoters which control gene expression and bacterial growth, have been used to regulate biological functions^4,6^.

### External triggering

Several bacterial promoters that respond to specific chemicals have been developed to ensure the expression of payloads in a dose-dependent and timely manner. Chemical inducers control the timing of payload production and its release within the tumor. However, the potential toxicity of some inducers and the risk of target gene leakage limit the use of this approach^10–12^. The P_*BAD*_ promoter induced by L-arabinose in the pBAD system has been used to express antitumor payloads such as cytolysin A (ClyA) and an immunomodulatory flagellin subunit (FlaB), or to promote cellular invasion of engineered *Salmonella*^4,12,13^. An alternative strategy is to use a bidirectional P_*tet*_ promoter (P_*tetA*_ and P_*tetR*_), which is induced by a class of FDA-approved antibiotics (doxycycline [Doxy] or tetracycline), to ensure the proportional expression of multiple payloads^14^.

Physical signals have been used to control the activity of cellular processes in living organisms. Unlike chemical inducers, physical signals such as visible light and heat are non-invasive, non-toxic, and artificially controllable; they can therefore be used to induce gene expression in a more spatiotemporal manner than conventional regulatory strategies, rendering them more suitable for BCiT^15^. Optogenetics is a combination of optical and genetic techniques in which light-sensitive proteins control genetic systems specific to a particular cellular activity^15^. Because visible light cannot pass deep into the tissues, the delivery of photons is an important issue with respect to optogenetics for in vivo applications. To overcome this, an upconversion optogenetic system is being developed to convert near-infrared light to visible blue light^15,16^. Thermal regulation has been more commonly used in BCiT than optogenetics. For example, the introduction of a gene circuit encoding an orthogonal heat switch into the *E. coli* Nissle 1917 (EcN) strain expressing tumor necrosis factor-α (TNF-α) or melanin, triggered a strong antitumor immune response^17,18^. EcN was engineered by the introduction of a temperature-activated genetic switch that induced the expression of therapeutic agents such as immune checkpoint inhibitors in response to hypothermia triggered by a brief application of focused ultrasound (FUS)^19^.

### Internal triggering and quorum sensing

The genetic circuit can be designed in a way that causes bacteria to specifically produce therapeutic agents in response to tumor-specific internal triggers, such as a certain pH, oxygen concentration, or glucose gradient in the TME^4,6,20^.

Because bacteria specifically colonize tumors and preferentially proliferate in the TME, the use of the bacterial quorum-sensing (QS) system can facilitate the release of therapeutic payloads into tumors without causing damage to healthy tissues^6,21,22^. In recent studies by Gurbatri et al. and Chowdhury et al., *E. coli* were engineered to express QS-responsive molecules such as acyl-homoserine lactone (AHL)^6^, which induced bacterial lysis and the subsequent release of therapeutic payload when the bacterial population density reaches a specific threshold within the TME.

### Synthetic logic circuit systems

A combination of gene circuits can be designed to respond to multiple input signals and prevent leaky expression caused by a single gene circuit. Boolean logic gates (i.e., AND, OR, and XOR) are suggested to generate output via multiple input signals in combination, based on digital logic rules. One of the simplest gates is the AND logic gate, in which all input signals are required to switch on the output. Shong et al. engineered *E. coli* harboring the QS-based AND logic gate, which controls gene expression by sensing both AHL and an additional input signal such as IPTG or anhydrotetracycline (aTc)^23^.

## Engineering live bacteria for cancer immunotherapy

**Fig. 1:**
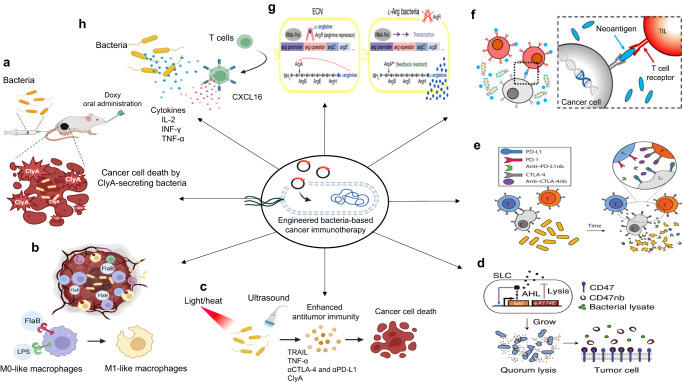
Engineered bacteria-based cancer immunotherapy. **a**, **b** In the presence of chemical molecules such as Doxy or l-arabinose, bacteria secrete ClyA to kill cancer cells, or FlaB to reprogram the TME, to increase recruitment of antitumor M1-like macrophages through TLR-4 and TLR-5 signaling^4,13,14^. **c** Other potential approaches include physical factors such as light, heat, or focused ultrasound, which can be used to stimulate bacteria to deliver immunotherapeutics into the TME^4,6,17,19,35,36^. **d**, **e** Bacteria have been genetically modified with the QS system, which in this case is based on the AHL autoinducer. In this system, AHL is produced via the luxI promoter and controlled expression of lysE, resulting in quorum-mediated lysis and intratumoral release of drugs such as CD47-blocking nanobodies or PD-L1- and CTLA-4-blocking nanobodies. Reproduced with permission from ref. ^26^, reprinted with permission from Springer Nature, ref. ^33^, and the American Association for the Advancement of Science (AAAS). **f** Tumor-infiltrating T cells stimulated by cleaved neoantigens detect and kill tumor cells. Reprinted with permission from ref. ^27^ (copyright 2021, American Chemical Society). **g**, **h** Engineered bacteria can also stimulate adaptive immune responses against tumors by secreting cytokines, chemokines, or other immunomodulatory cargoes to recruit and activate TILs in the TME^6,21,34^. Reproduced with permission from ref. ^34^ and reprinted with permission from Springer Nature. The image was created using BioRender.com.

Tumor-colonizing bacteria can effectively activate innate and adaptive immune systems through surface components and intracellular molecules^4^. Together with their intrinsic immunostimulatory nature, expression/release of payloads can enhance antitumor immune responses; thus, the bacteria can be controlled precisely using synthetic biology approaches, ultimately improving overall therapeutic efficacy through recruitment and activation of the innate and adaptive immune systems (Fig. 1)^6^.

Live bacteria act as strong immune adjuvants to recruit and stimulate innate immune cells such as monocytes, neutrophils, and macrophages in tumors. These activated immune cells produce inflammatory cytokines such as interleukin-1β (IL-1β), TNF-α, and interferon-γ (IFN-γ)^4,6^. For instance, ref. ^13^. engineered the ∆ppGpp strain to secrete heterologous FlaB, which led to an increase in the size of the M1-like proinflammatory macrophage population, while reducing M2-like anti-inflammatory macrophage numbers.

Bacteria can also be used to enhance the immunogenicity of “cold tumors”, which are characterized by a lack of cytotoxic T cell infiltration, by secreting cytokines or chemokines to attract T cells to the tumor site. The in situ production of potent immunostimulatory molecules, such as IL-2, IFN-γ, TNF-α, and chemokine C-X-C motif ligand 16 (CXCL16), increases anticancer immune responses^4,6,24^. In a recent phase I clinical trial, the oral administration of *Salmonella* expressing human IL-2, a potent immunostimulatory cytokine, significantly increased the numbers of circulating natural killer (NK) and NKT cells in 22 patients with metastatic gastrointestinal cancer^25^.

Advances in synthetic biology have further improved the efficacy of BCiT. For example, a synchronized lysis gene circuit, which enables the controlled release of nanobodies specific for CD47, PD-L1, or CTLA-4, was developed to program the EcN strain^6^. CD47 interacts with thrombospondin-1 and signal regulatory protein α (SIRPα) to facilitate escape from macrophage-mediated immune surveillance by blocking phagocytotic mechanisms^26^. A non-pathogenic *E. coli* strain harboring the QS system can be engineered to deliver locally a CD47 nanobody as an immunotherapeutic payload to enhance antitumor immunity^26^. Moreover, a synthetic biology approach was used to engineer l-arginine-producing EcN, which increased the typically low l-arginine concentration within the TME and promoted an antitumor T cell response^6,21^.

The presentation of mutated tumor-derived antigens (neoantigens) by professional antigen-presenting cells induces potent tumor-specific T cell activation^27^. Hyun et al. used this principle to engineer a ∆ppGpp strain expressing multiple neoantigen peptides on the bacterial outer membrane. These peptides were specifically cleaved by matrix metalloproteinases (MMP) expressed on tumor cells^6^. The subsequent release of neoantigens from bacterial cells at the tumor site enhanced the antitumor immune response by activating neoantigen-specific T cells.

## Clinical trials and future outlooks

Significant progress has been made in the engineering of bacteria for BCiT. For instance, Bacillus Calmette-Guérin (BCG), a live attenuated *Mycobacterium tuberculosis* vaccine, has been effectively used for high-risk, non-muscle-invasive bladder cancer^28,29^. Furthermore, the National Institutes of Health’s website “clinicaltrials.gov” lists over 50 completed clinical trials of live bacteria, including *Salmonella* (*n* = 13), *Listeria* (*n* = 32), *Clostridium* (*n* = 4), *Bifidobacterium* (*n* = 2), and *Yersinia* (*n* = 1), for the treatment for various malignancies^3,30^. Indeed, BCiT with live bacteria is no longer limited to preclinical studies.

However, despite on-going clinical translational activities, drug development using live bacteria presents particularly difficult challenges both for investigators and regulatory authorities. First, the use of live bacteria in BCiT cannot be regulated in the same way as conventional drugs, while heating or filtering cannot be used to sterilize live bacteria. If the bacteria are to be used as therapeutic agents, it is crucial to maintain axenic cultures and eliminate potentially harmful substances, such as bacterial pathogens^9^. Moreover, the viability of therapeutic bacteria should be guaranteed (without batch-to-batch variation), regardless of any upscaling (to facilitate production on an industrial scale), packaging, shipping, or storage methods^31^. Second, determining the appropriate dose and schedule for BCiT is also challenging because bacteria may not follow conventional pharmacokinetics and may have a different dose-response relationship than other drugs. Effective doses may be related more closely to the quality of the target tumor rather than to the bacterial dose administered^9^. Third, pathogenic infection is a potential risk factor. Although the live bacteria used in BCiT have low levels of virulence and can be eradicated using antibiotics if required, they still pose a potential risk to immunocompromised patients, as well as to patients with abscesses, artificial joints/heart valves, or a recent history of radiation therapy use^9^. Thus, these patients may need to be excluded from BCiT clinical trials in the first instance. Finally, it is crucial to exclude bacterial strains carrying antibiotic resistance genes to avoid the risk of horizontal gene transfer^9^.

The currently available FDA guidance documents related to BCiT are “Microbial Vectors used for Gene Therapy” (September 2016)^31^ and “Preclinical Assessment of Investigational Cellular and Gene Therapy Products” (November 2013)^32^. Moreover, it is recommended that potential sponsors of investigational new drugs contact the FDA to obtain additional guidelines before submission.

In conclusion, the results of clinical trials will determine the practical application of BCiT in the clinic. Several engineered live bacterial therapeutics are currently entering early or mid-stage clinical development and may provide the proof of concept needed to determine the future of this new class of therapeutic agents. Upon reaching these milestones, developers of engineered bacterial therapeutics will be able to establish optimal safety, administration, and manufacturing processes, as well as determine how these agents can be effectively combined with existing therapies. Optimizing the application of this new category of drugs will address the significant unmet needs of patients. However, regulatory hurdles must be overcome to make this a reality.
